# Role of regulatory T cells in murine model of liver fibrosis resolution

**DOI:** 10.1186/1753-6561-6-S4-O42

**Published:** 2012-07-09

**Authors:** X Lim, P Ramachandran, JP Iredale

**Affiliations:** 1 College of Medicine and Veterinary Medicine, University of Edinburgh, MRC Centre of Inflammation Research, UK

## Introduction

Liver fibrosis is a chronic inflammatory condition that afflicts many people. While much is known about the role of macrophages subpopulations in liver fibrosis resolution, little is known about the role of regulatory T cells (Tregs) in the process. Because Tregs are known to dampen inflammatory responses, we hypothesize that Tregs mediate the switch from fibrosis to resolution of liver fibrosis by interacting with macrophages.

## Methods

Using various Treg and macrophage conditional ablation models, we aimed to elucidate the relationship between Tregs and pro-resolution macrophages. The 5 models used are: Foxp3-DT Treg depletion, CD11b-DT macrophage depletion, MMP-12 knock-outs, liposome-induced pro-resolution macrophage activation and liposome clodronate macrophage depletion. All mice were injected with carbon tetrachloride for 4 weeks and allowed to recover.The livers were harvested at various time points, fixed and stained for fibrillar collagen, hepatic stellate cells (HSCs), Tregs and macrophages. Mann-Whitney statistical test was used and values of p<0.05 were considered significant.

## Results

We established that Foxp3-DT is a novel viable method of Treg depletion (p<0.05). Treg depletion resulted in failure of fibrosis remodelling as shown by picrosirius red staining and morphometry (p<0.05). CD11b pro-resolution macrophage depletion caused an increase in number of Tregs during resolution of liver fibrosis (p<0.05) while liposome-induced activation of pro-resolution macrophages caused a decrease in Tregs during resolution of liver fibrosis (p<0.05). We also established that MMP-12 is a marker of pro-resolution macrophages and observed that MMP-12 knock-outs had increased Tregs during resolution of liver fibrosis (p<0.05). However, we also observed that liposome clodronate macrophage depletion resulted in lower number of Tregs.

## Conclusions

We conclude that Tregs are necessary for fibrosis resolution and that there is an inverse relationship between Tregs and pro-resolution macrophages. We propose that this may be due to negative feedback signals from pro-resolution macrophages and/or feed forward signals from inflammatory macrophages recruiting Tregs. Similar observations with MMP-12 knock-out mice suggest this process may be mediated by MMP-12.

